# The Role of Oncogenic Viruses in Head and Neck Cancers: Epidemiology, Pathogenesis, and Advancements in Detection Methods

**DOI:** 10.3390/microorganisms12071482

**Published:** 2024-07-19

**Authors:** Pinelopi Samara, Michail Athanasopoulos, Stylianos Mastronikolis, Efthymios Kyrodimos, Ioannis Athanasopoulos, Nicholas S. Mastronikolis

**Affiliations:** 1 Children’s Oncology, Unit Marianna V. Vardinoyannis-ELPIDA, Aghia Sophia Children’s Hospital, 11527 Athens, Greece; 2 Department of Otolaryngology, University Hospital of Patras, 26504 Patras, Greece; miathanasopoulos@gmail.com (M.A.); nmastr@otenet.gr (N.S.M.); 3 Department of Ophthalmology, Medical School, University of Patras, 26504 Patras, Greece; mastronikst@gmail.com; 4 1st Department of Otorhinolaryngology, Hippocration Hospital, University of Athens, 11527 Athens, Greece; timkirodimos@hotmail.com; 5 Otolaryngology-Head & Neck Surgery, Athens Pediatric Center, 15125 Athens, Greece; athanasopoulosj@hotmail.com

**Keywords:** head and neck cancer, oncogenic viruses, human papillomavirus, Epstein–Barr virus, human herpesvirus 8, Merkel cell polyomavirus, hepatitis B, hepatitis C

## Abstract

Head and neck cancers (HNCs) constitute a wide range of malignancies originating from the epithelial lining of the upper aerodigestive tract, including the oral cavity, pharynx, larynx, nasal cavity, paranasal sinuses, and salivary glands. Although lymphomas affecting this region are not conventionally classified as HNCs, they may occur in lymph nodes or mucosa-associated lymphoid tissues within the head and neck. Oncogenic viruses play a crucial role in HNC onset. Human papillomavirus (HPV) is extensively studied for its association with oropharyngeal cancers; nevertheless, other oncogenic viruses also contribute to HNC development. This review provides an overview of the epidemiology, pathogenesis, and advancements in detection methods of oncogenic viruses associated with HNCs, recognizing HPV’s well-established role while exploring additional viral connections. Notably, Epstein–Barr virus is linked to nasopharyngeal carcinoma and lymphomas. Human herpesvirus 8 is implicated in Kaposi’s sarcoma, and Merkel cell polyomavirus is associated with subsets of HNCs. Additionally, hepatitis viruses are examined for their potential association with HNCs. Understanding the viral contributions in the head and neck area is critical for refining therapeutic approaches. This review underlines the interaction between viruses and malignancies in this region, highlighting the necessity for ongoing research to elucidate additional mechanisms and enhance clinical outcomes.

## 1. Introduction

Head and neck cancers (HNCs) constitute a diverse spectrum of malignancies originating from the upper aerodigestive tract, encompassing regions such as the oral cavity, pharynx, larynx, nasal cavity, paranasal sinuses, and salivary glands. About 90% of all HNCs are squamous cell carcinomas [1]. Thyroid cancer, skin cancers affecting the head and neck, and ear cancers are typically managed separately from traditional HNCs, even though they are anatomically situated in this vicinity. Additionally, lymphomas, while not conventionally classified as HNCs, can also affect the head and neck region [2].

The clinical management of HNCs poses significant challenges due to their complex etiology, heterogeneous nature, and varied treatment responses [3]. Despite advancements in diagnostics and therapies, HNCs remain a global burden, with over 650,000 new cases and 330,000 deaths annually [4], underscoring the need for deeper insights into their underlying mechanisms.

Among the multifaceted contributors to HNC development, oncogenic viruses have emerged as pivotal factors, profoundly influencing both the initiation and progression of these malignancies [5]. There are currently seven known human oncogenic viruses, which include five DNA viruses: human papillomavirus (HPV), Epstein–Barr virus (EBV), Kaposi’s sarcoma herpesvirus or human herpesvirus 8 (KSHV or HHV-8), Merkel cell polyomavirus (MCPV), and hepatitis B virus (HBV). Furthermore, there are two RNA viruses: hepatitis C virus (HCV) and human T-lymphotropic virus type 1 (HTLV-1) [6]. HTLV-1 primarily associates with T-cell malignancies such as adult T-cell leukemia/lymphoma and HTLV-1-associated myelopathy/tropical spastic paraparesis, with minimal direct involvement in HNCs [7]. Therefore, this review focuses on the other six oncogenic viruses that demonstrate a more direct association with HNCs.

These viruses induce cellular transformation through various mechanisms, including interference with cell cycle regulation, evasion of immune surveillance, and promotion of genomic instability [8]. For instance, HPV integrates its DNA into host cells, leading to the overexpression of early oncogenes E6 and E7, which inactivate tumor suppressor proteins p53 and Rb, respectively [9]. The incidence of HPV-associated HNCs, particularly oropharyngeal squamous cell carcinoma (OPSCC), has risen significantly, attributing approximately 70% of oropharyngeal cancers in the United States to HPV, profoundly influencing clinical management and patient outcomes [10]. Similarly, EBV is implicated in nearly all nasopharyngeal carcinomas and a significant proportion of head and neck lymphomas, illustrating the intricate viral interactions within the tumor microenvironment [11].

Distinct HNC subtypes exhibit unique viral profiles and molecular signatures, reflecting diverse biological behaviors and responses to treatment [12]. Understanding the interplay between oncogenic viruses and host factors is essential for developing targeted therapeutic strategies that effectively combat HNC progression while minimizing treatment-related morbidity. Advances in detection methods, such as highly sensitive polymerase chain reaction (PCR) assays, next-generation sequencing (NGS) [13], and advanced imaging [14], have revolutionized the identification and characterization of viral involvement in HNCs. These technologies enable early diagnosis, improved prognostication, and personalized treatment approaches, ultimately enhancing patient outcomes.

The goal of this review is to provide a comprehensive overview of the role of oncogenic viruses in HNCs (Table 1), focusing on their impact on disease development and progression. By synthesizing current knowledge on HPV, EBV, and other relevant viruses, we aim to elucidate their diverse roles across different HNC subtypes. Furthermore, we will discuss recent advancements in viral detection technologies and their potential to enhance early diagnosis and refine clinical management strategies. Understanding the intricate relationship between oncogenic viruses and HNCs is crucial for guiding future research directions and optimizing diagnostic approaches in this challenging disease context.

## 2. Human Papillomavirus (HPV) in Head and Neck Cancers

The prevalence of HPV-associated HNCs varies widely, ranging from 20–80%. This significant variation is attributed to a multitude of factors, including geographic disparities, diverse demographic profiles, and the varying sensitivity and specificity of the methods employed for HPV detection [10]. These differences emphasize the complexity of understanding HPV’s impact on HNCs across different populations and the requirement for standardized diagnostic criteria to accurately assess and address this global health issue.

### 2.1. HPV’s Association with Oropharyngeal Cancers: Mechanisms Underlying HPV-Induced Carcinogenesis in the Oropharynx

HPVs are prevalent sexually transmitted infections affecting both sexes globally and are extensively reviewed in the literature. The well-established link between HPV infection and various cancers extends to penile, cervical, vaginal, vulvar, and HNCs, particularly oropharyngeal cancer [15]. Over 200 HPV genotypes have been categorized into low-risk and high-risk types, based on their oncogenic potential. According to the International Agency for Research on Cancer (IARC), twelve HPV types (16, 18, 31, 33, 35, 39, 45, 51, 52, 56, 58, and 59) are classified as carcinogenic to humans [16].

HPVs spread via direct sexual contact, facilitated by the long control region within their genome, which contributes significantly to genomic variation. The viral genome consists of E1, E2, E4, E5, E6, E7, and E8 genes responsible for replication and cellular transformation, as well as late genes for viral particle assembly [17]. HPV infects epithelial cells through microabrasions or small wounds, primarily targeting basal cells, where it initiates infection. This non-productive infection allows the virus to persist within the cellular environment over time [18]. The viral life cycle comprises acquisition, persistence, pre-cancer progression, and invasion stages, with persistent infection significantly increasing cancer risk. Therefore, understanding HPV’s capability to establish long-term infections is crucial beyond its pathogenic effects.

HPV maintains its genome in an episomal form, compartmentalizing gene expression across various cellular levels [19]. Upon entering basal epithelial cells, the virus minimally expresses E1 and E2 genes, exploiting the host cellular replication machinery. During the non-productive phase, HPV evades immune detection and expresses E6 and E7 genes alongside E1 and E2 in suprabasal cells, disrupting cell cycle regulators and leading to cell immortalization [20]. Early gene expression, particularly E4 and E5, supports viral genome amplification and new virion production [5].

In many cases, the immune system clears HPV infections acquired through sexual activity. However, when HPV integrates into the host genome, a critical step in HPV-induced cancer development occurs. Integrated HPV expresses unique oncogenes, affecting cell cycle control mechanisms and inducing genomic instability, ultimately leading to cancer progression [21]. HPV E6 degrades p53 via E6AP, inhibiting apoptosis, while E7 targets and degrades pRb, promoting abnormal cell proliferation. Additionally, E6 interacts with c-myc, elevating h-TERT levels, further contributing to cellular transformation and unlimited lifespan [5].

While the fundamental mechanisms of HPV infection and viral lifecycle are similar in cervical and oral epithelial cells, tissue-specific factors such as epithelial structure and microbiota composition, immune responses, and environmental influences contribute to differences in how HPV-associated cancers manifest and progress in these different anatomical sites. The precise factors facilitating the persistence of oral HPV infection remain elusive. Typically, HPV begins as a circular episomal form within cells before transitioning to a linear form and integrating into the host genome. Integrated HPV is commonly found in cervical cancer [22]. In HNCs, HPV integration occurs at multiple insertional breakpoints rather than at a single site [23]. Research on HNC cell lines has revealed that these breakpoints are associated with genomic instability, a key characteristic of virus-induced carcinogenesis. Integration leads to upregulated expression of HPV E6 and E7, further driving cellular transformation [24].

### 2.2. HPV Infection and Its Implications in Laryngeal Cancer

While HPV’s role in oropharyngeal and genital cancers is well-documented, its involvement in laryngeal cancer is less definitive. Although smoking and alcohol use remain the primary risk factors for laryngeal cancer, HPV is notably present in non-smokers and younger patients. Among various HPV subtypes, HPV-16 and HPV-18 are often detected in laryngeal cancer, with HPV-16 strongly linked to cancer development and prognosis [25].

A decade ago, Li et al. [26] conducted a systematic review and meta-analysis to examine the association between HPV infection and laryngeal cancer. Their review of 55 studies revealed an overall HPV prevalence of 28.0% in laryngeal cancer tissues, with 26.6% attributed to high-risk HPV types, notably HPV-16 at 19.8%. A meta-analysis of 12 case–control studies confirmed a significant association between HPV infection and laryngeal squamous cell carcinoma (LSCC), particularly with HPV-16. Three years later, Gama and colleagues performed a thorough meta-analysis investigating the prevalence of HPV in LSCC through an exhaustive review of 179 studies encompassing 7347 cases. Their results revealed that HPV was present in 25% of cases, with notable variability primarily linked to geographic factors rather than the specific methods of HPV detection [27]. This underscores the significant influence of geographic origin on HPV’s impact on the epidemiology of laryngeal cancer.

One year later, Erkul and colleagues investigated the presence and potential prognostic role of HPV in LSCC. They detected HPV DNA in 26.02% of cases and observed a slightly higher 3-year survival rate among HPV-positive patients compared to those without HPV, although these differences were not statistically significant. The study also highlighted an increasing prevalence of high-risk HPV-16 in these cancers compared to previous reports [28]. Despite these findings, the authors concluded that HPV may not be sufficiently reliable as a biomarker for diagnostic or prognostic purposes in LSCC.

### 2.3. The Role of HPV Vaccination in Prevention Strategies for HPV-Associated HNCs

HPV vaccination has demonstrated high effectiveness in preventing HPV infections, thereby reducing the incidence of HPV-related cancers, particularly those associated with oncogenic HPV types such as HPV-16 and HPV-18 [29,30]. While direct evidence linking HPV vaccination to preventing HNCs is limited compared to cervical cancer, ongoing research suggests that HPV vaccination contributes to a decrease in HPV-related HNC diagnoses. Studies are also exploring the potential secondary effects of HPV vaccination on HPV prevalence in other anatomical sites, with significant efficacy demonstrated against oral and oropharyngeal HPV infections [31]. Vaccination has shown promise in reducing oral HPV infections and HPV-associated precancerous lesions in the oropharynx, offering potential long-term cancer prevention benefits [32].

In addition to directly preventing HPV infection, HPV vaccination contributes to herd immunity, lowering overall HPV prevalence in the population and indirectly protecting unvaccinated individuals. This collective impact has the potential to significantly reduce the incidence of HPV-associated HNCs over time. Chaturvedi et al. [33] reported a notable 37% decrease in vaccine-type oral HPV prevalence among unvaccinated men aged 18 to 59 years in the United States from 2009 to 2016, coinciding with increasing HPV vaccination rates among females. However, non-vaccine-type oral HPV infections showed no change in prevalence among both men and women, underscoring the targeted efficacy of HPV vaccination against specific HPV types. These findings stress the public health benefits of HPV vaccination in reducing oral HPV infections and highlight the importance of ongoing surveillance and expanded vaccination efforts. In 2020, these outcomes prompted the FDA to expand approval of Merck’s HPV 9-valent vaccine (Gardasil 9) to include prevention of oropharyngeal and other HNCs caused by HPV types 16, 18, 31, 33, 45, 52, and 58, using accelerated pathways [34].

Several clinical trials are currently evaluating the efficacy of HPV vaccines in preventing HNCs. A significant study by Giuliano et al. focuses on assessing the 9-valent HPV vaccine’s potential to prevent oral persistent infections, which are closely linked to HPV-related HNCs. This phase III trial involves a substantial cohort of 6000 men across international sites and employs a rigorous design to evaluate vaccine efficacy, immunogenicity, and safety profiles. It is expected to provide critical insights into preventive strategies against HPV-related head and neck diseases [35].

Despite the proven effectiveness and safety of HPV vaccines, their uptake remains suboptimal in many regions globally. Barriers to vaccination include safety concerns, limited access to healthcare, and insufficient awareness regarding the link between HPV and HNCs. Enhancing vaccination rates through education, targeted public health campaigns, and integrating vaccination programs into routine healthcare services are essential steps for maximizing the preventive potential of HPV vaccines.

## 3. Beyond HPV: Emerging Oncogenic Viruses in HNCs

### 3.1. Epstein–Barr Virus (EBV): Its Role in Nasopharyngeal and Other Head and Neck Cancers

EBV, also known as human herpesvirus type 4 (HHV-4), was first identified in 1964 in a Burkitt lymphoma cell line [36] and is undoubtedly the most oncogenic herpesvirus. It infects approximately 90–95% of the adult human population globally, establishing a lifelong latent infection, primarily transmitted through saliva [37]. This widespread distribution highlights its significant role in various cancers, including nasopharyngeal carcinoma, Burkitt and Hodgkin lymphomas, and gastric carcinoma. EBV is categorized as a Group I carcinogen by the IARC [11,38].

EBV employs a “hit and run” strategy, manipulating host epigenetic mechanisms to initiate oncogenic pathways even after the virus is cleared. This reprogramming results in heritable changes in gene expression, influencing cancer progression independently of ongoing viral presence [39]. EBV’s oncogenic effects are mediated through latent viral proteins such as EBV nuclear antigens (EBNAs) and latent membrane proteins (LMPs), which disrupt cellular functions, promote proliferation, and inhibit apoptosis [40]. Its genome comprises linear double-stranded DNA encoding about 80 genes and surface glycoproteins, including terminal repeats critical for episome formation [41]. The virus can enter lytic or latent phases affecting B-cells and epithelial cells. During latency, EBV transforms cells by manipulating cellular pathways via genes like EBNA1 and LMP1 [42].

In HNCs, particularly nasopharyngeal and oral squamous cell carcinomas (OSCC), EBV’s latent proteins, like LMP1, mimic CD40 and tumor necrosis factor receptors, stimulating uncontrolled proliferation [43]. Despite advances in understanding its role in B-cell malignancies, EBV’s mechanisms in epithelial cell cancers remain less clear, with ongoing research indicating latent phase existence even in healthy epithelial cells [44].

EBV has also been detected in laryngeal carcinoma cells, suggesting it is a potential risk factor. Despite its low prevalence in laryngeal cancer and detection challenges, EBV appears to drive tumor development. Brichácek et al. were the first to identify the EBV genome and latent protein EBNA in malignant laryngeal carcinoma cells [45]. A systematic review and meta-analysis by de Lima et al. suggested that EBV might act as a risk factor or cofactor in laryngeal carcinoma development and progression [46]. However, the exact role of EBV in LSCC remains controversial due to difficulties in detecting the virus and its low prevalence in this cancer type. Continued research into EBV–host interactions and the development of targeted therapies offer the potential for reducing EBV-associated malignancy burden in the future.

### 3.2. Human Herpesvirus 8 (HHV-8): The Second Most Prevalent Oncogenic Herpesvirus

HHV-8, also referred to as Kaposi’s sarcoma-associated herpesvirus (KSHV), is a notable oncogenic pathogen associated with HNCs, first identified in 1994. The virus is predominantly linked to Kaposi’s sarcoma (KS), a malignancy commonly affecting the mucosal tissues of the mouth and throat, notably prevalent in HIV-positive individuals due to their compromised immune systems [47]. Unlike other human herpesviruses, HHV-8 exhibits a distinct geographical distribution, with the highest seroprevalence rates observed in Mediterranean regions and Africa, and lower rates elsewhere [48].

HHV-8 establishes latent infections in endothelial cells and exerts oncogenic effects through viral proteins that disrupt cellular regulatory mechanisms, facilitating uncontrolled cell proliferation and survival. HHV-8 encodes several transforming proteins, including viral G protein-coupled receptor, viral interleukin-6, and latency-associated nuclear antigen (LANA) [49]. These proteins activate signaling pathways such as MAPK, PI3K/Akt, and NF-κB, leading to cell proliferation, angiogenesis, and inhibition of apoptosis. The ability of the virus to modulate the host immune response further enhances its oncogenic potential by evading immune detection and creating a microenvironment conducive to tumor development [50]. Beyond KS, HHV-8 is implicated in primary effusion lymphoma (PEL) and multicentric Castleman disease (MCD), both of which can involve the head and neck region. PEL is a rare, aggressive B-cell lymphoma that predominantly occurs in body cavities but can also be present in the oral cavity [51]. MCD is a lymphoproliferative disorder that can manifest with lymphadenopathy in the head and neck, with HHV-8 found in the lymphoid tissues, driving disease progression [52]. Güvenç et al. explored HHV-8 in 47 patients with LSCC, alongside HPV, and detected HHV-8 in five of these patients. One case was found to have both HHV-8 and HPV DNA, suggesting that HHV-8, in addition to HPV, might contribute to laryngeal carcinogenesis [53].

## 4. Other Potentially Oncogenic Herpesviruses Influencing Head and Neck Cancers

Human herpesvirus 1 (HHV-1), or Herpes simplex virus 1 (HSV-1), primarily causes oral herpes through latent infections in nerve cells that can periodically reactivate, leading to recurrent oral lesions. HSV-1 has been investigated for its potential role in oral cancer pathogenesis, hypothesized to contribute through chronic inflammation and inducing genetic mutations in host cells. Despite the detection of viral markers in oral cancer tissues, research on HSV-1’s association with HNCs has yielded conflicting findings [54,55]. In a recent study, Von Stebut and colleagues sought to evaluate the link between HSV infections and HCNs using data from TriNetX. Their retrospective analysis included 249,272 patients, comparing those with and without HSV infections. The study identified a significant association between HSV infection and lip cancer, demonstrating a notable hazard ratio of 1.17 across all HNCs [56]. These discoveries highlight the potential clinical significance of seemingly benign HSV infections as a novel factor for risk stratification in lip, oral cavity, and pharynx cancers.

Other herpesviruses, including Cytomegalovirus (CMV), have also been investigated for their potential roles in HNCs. While their direct oncogenic roles remain unclear, CMV and similar viruses may contribute to cancer development through mechanisms such as chronic inflammation, immune modulation, and interactions with other oncogenic factors. While CMV is not typically considered oncogenic, it has been implicated in various cancers and studied for its potential anti-tumor effects in clinical trials involving dendritic cell vaccines and CMV-specific cytotoxic T cells. Trivic et al. [57] recently directed a global study exploring the association between human CMV infection and head and neck tumors. Their analysis across 73 countries identified a pro-oncogenic link with nasopharyngeal carcinoma and a protective effect with tumors of the lip/oral region and salivary glands. However, this protective effect did not persist after adjusting for other variables in thyroid neoplasia and hypopharyngeal tumors. No significant association was observed between CMV and laryngeal cancer. These findings suggest that CMV may exert tissue-specific effects that could inform future therapeutic strategies for certain HNCs, accentuating the need for further research to validate these findings and elucidate underlying mechanisms.

The involvement of herpesviruses other than HHV-1 in HNCs is not fully explained. HHV-2 (HSV-2), for instance, has shown no significant link to oral cancer, and HSV-1/HSV-2 infections were not significantly associated with HNCs. HHV-3 (Varicella zoster virus) has not been confirmed as an oncogenic factor but has been linked to higher cancer rates in individuals with herpes zoster. HHV-6 and HHV-7, detected in salivary glands, exhibit potential tumorigenic properties and the ability to transactivate viruses, implying a role in oral carcinogenesis, although conclusive evidence is lacking. These findings are comprehensively reviewed in the elegant review by Wołacewicz et al. [58].

Continued exploration of these viruses in HNCs holds promise for improving diagnostic, preventive, and therapeutic approaches, ultimately enhancing patient outcomes and quality of life.

## 5. Merkel Cell Polyomavirus (MCPV) and Its Role in Head and Neck Cancers

Several studies are in progress to elucidate the role of human polyomaviruses, like MCPV, in the pathogenesis of HNCs. MCPV is associated with Merkel cell carcinoma, a rare and aggressive neuroendocrine skin carcinoma. In addition, it has been implicated in the development of HNCs, particularly oral and pharyngeal carcinomas. Similar to EBV infection, MCPV infection often occurs asymptomatically during childhood and can persist on the skin of healthy adults [59].

Mohebbi et al. [60] investigated MCPV in Iranian patients with head and neck squamous cell carcinomas (HNSCC). They examined 50 HNSCC biopsy samples and found MCPV DNA in 16.0% of cases, with a higher viral load in stage III tumors. The study suggests that MCPV infection may affect only a subset of HNSCC cases, emphasizing the need for further research. A study by Muñoz et al. [61] revealed that MCPV was detected in 12.5% of HNSCCs analyzed in Chilean patients. In contrast, BK human polyomavirus (BKPV) and JC human polyomavirus (JCPV) were rarely found, with only one case of BKPV identified and none of JCPV in the HNSCC samples. Importantly, MCPV was not detected in oral brushes from subjects without cancer, suggesting a potential association of MCPV specifically with HNSCCs. Estalkhi et al. [62] conducted a study in Northern Iran examining MCPV prevalence in 114 oral cavity biopsies, comparing cancerous (OSCC, dysplasia) and non-cancerous lesions [oral lichen planus (OLP), and oral irritation fibroma (OIF)]. They found MCPV DNA in 20% of OSCC samples, 21.4% of dysplasia samples, 24.1% of OLP samples, and 30.6% of OIF samples. These results suggest MCPV may be involved in both cancerous and non-cancerous oral lesions, indicating its potential role in oral cavity pathogenesis warranting further investigation. Overall, the existing literature suggests a need for further investigation to fully understand the role of MCPV in the development of HNCs.

## 6. Human Bocavirus (HBoV) and Head and Neck Cancers: Exploring Its Role in Tonsil Squamous Cell Carcinomas

Discovered in 2005, Human Bocavirus (HBoV) belongs to the Bocaparvovirus genus of the Parvoviridae family and is associated with respiratory and gastrointestinal infections, often persisting subclinically in infected cells [63]. This persistence is particularly evident in cancer patients, where HBoV DNA has been detected in approximately 20% of colorectal and lung cancer tissues, and is more prevalent in the serum of cancer patients compared to healthy individuals [64]. HBoV has also been implicated in tonsil squamous cell carcinomas (TSCC). Höpken et al. [65] found that 60.19% of TSCC samples tested positive for HBoV DNA, with 66% also positive for HPV DNA. Fluorescence in situ hybridization analysis indicated both single and co-infections of HBoV and HPV. Notably, 22 out of 62 HBoV-positive tumors were HPV-negative, suggesting HBoV’s potential independent role in carcinogenesis.

Supporting HBoV’s oncogenic potential, whole transcriptome analysis revealed significant upregulation of pathways associated with neoplasia and tumorigenesis in HBoV-infected cells. Specific transcripts regulated by HBoV influence these pathways, potentially contributing to tumor development, while pathways related to necrosis, apoptosis, and cell death were downregulated, further implicating HBoV in cancer progression [66]. Ivaska et al. [67] explored persistent HBoV1 infection in tonsillar tissues of 143 tonsillectomy patients, detecting it in 17% of cases. They observed that persistent HBoV1 infection correlated with diminished T-helper17 and T-regulatory immune responses, indicated by suppressed transcription factors RORC2 and FOXP3. Moreover, HBoV1-DNA loads negatively correlated with IFN-λ family cytokines and IL-13, suggesting a suppressive role of HBoV1 in immune modulation.

These findings suggest that HBoV may contribute to TSCC development, either as a cofactor or through a tumor tropism mechanism. The chronic inflammation and prolonged latency periods observed in HPV infections may parallel those seen in chronic HBoV infections, suggesting similar mechanisms in oncogenesis. Further research is crucial to fully comprehend HBoV’s pathophysiology, its interactions with cells and other viruses, and its potential as an oncogenic agent.

## 7. Epidemiology and Clinical Impact of Hepatitis Viruses in Head and Neck Cancers

Hepatitis B (HBV) and C (HCV) viruses are well-established carcinogens, notably associated with hepatocellular carcinoma [68] and non-Hodgkin lymphoma [69]. Furthermore, they are increasingly recognized for their role in elevating the risk of cancers outside the liver. Interest has been growing in exploring the impact of hepatitis virus infections on the development of HNCs [70,71].

HBV is a partially double-stranded DNA virus comprising genes that encode essential proteins for viral function, including surface antigens, core proteins, viral DNA polymerase, and replication proteins. Similar to EBV, HBV exhibits dual tropism for infecting naïve B-cells and hepatocytes. Following host cell entry, HBV’s genome translocates to the nucleus, initiating viral RNA transcription facilitated by DNA polymerase with reverse transcriptase activity [72]. HCV is a single-stranded positive-sense RNA virus with a genome encoding structural proteins and envelope glycoproteins. HCV exhibits triple tropism, infecting naïve B-cells, hepatocytes, and salivary gland cells. Upon infecting host cells, HCV activates pathways promoting malignancy by disrupting cell cycle regulation and inhibiting apoptosis [73]. Although the precise mechanism of HCV-induced head and neck carcinogenesis remains unclear, parallels can be drawn with HPV infection. HCV proteins like NS3 and NS5A interfere with cell cycle regulation by targeting proteins such as p53 and Rb for degradation [74].

Donà et al. [75] organized a retrospective case–control study to explore the association between chronic hepatitis B and C infections and HNSCC. They found that HNSCC patients were significantly more likely to have chronic hepatitis B and hepatitis C infections compared to cancer-free controls. These findings suggest a positive association between chronic hepatitis B and C infections and HNSCC, highlighting the need for further research to establish causality and the potential benefits of early detection for improved patient outcomes. Nayyar’s study in Indian populations [76] and Komori’s study in Japanese populations [77] reported consistent findings among Asian populations. Komori conducted a retrospective case–control study investigating the association between hepatitis B core (HBc) antibody positivity, a marker of prior HBV infection, and HNC. Analyzing data from 512 HNC and 495 non-HNC patients treated between 2008 and 2017, the study revealed a significant link between HBc antibody positivity and increased HNC risk. Smoking and a history of cancer were also identified as significant risk factors for HNC [77]. Despite limitations such as potential selection bias and incomplete data on alcohol consumption, these findings suggest that HBV infection may contribute to HNC pathogenesis in the Japanese population, highlighting the necessity for further research into the underlying biological mechanisms.

Conversely, a study by Su et al. [78] 12 years ago found that chronic HCV infection significantly increases the risk of oral cavity cancer. Using nationwide cohort data from Taiwan, they reported a 2.28-fold higher incidence of oral cavity cancers in HCV-infected patients compared to those without viral hepatitis. Adjusted for demographic factors, HCV infection alone showed a 90% higher risk of oral cavity cancer, particularly pronounced in adults aged 40–49 years. In contrast, HBV infection alone or HBV/HCV dual infections did not show significant associations with oral cavity cancer risk. Moreover, the meta-analysis by Borsetto et al. [79] synthesized evidence from eight observational studies, highlighting a significant association between chronic HCV infection and increased risk of HNSCC. Their conclusions revealed elevated risk ratios for oral cavity, oropharynx, and larynx cancers among HCV-infected individuals. Although the association with hypopharyngeal cancer was suggestive but not statistically significant due to limited data, these results underscore the importance of early surveillance for HNSCC in patients with chronic HCV infection and consider HCV screening in HNSCC clinical management. Hung et al. [80] carried out a case–control study involving 5603 HNC patients and 16,809 matched controls to investigate HBV and HCV infections. They found a higher prevalence of HBV among HNC cases (9.0%) compared to controls (7.6%). Similarly, HCV prevalence was elevated in HNC cases. Notably, oropharyngeal cancer patients showed a significantly higher likelihood of HCV infection compared to controls.

A meta-analysis by Tan et al. [81] showed a significant positive association between HBV infection and HNC. Their findings, based on a comprehensive review of 13 studies involving 58,006 HNC patients, indicated that HBV infection was correlated with an increased risk of HNC. Subgroup analyses further highlighted significant associations with oral cancer and nasopharyngeal carcinoma. Interestingly, Lai et al. [82] examined how HBV infection impacts survival in HNSCC patients. Analyzing 1015 patients, they found HBV-positive individuals (12.3% of the cohort) had higher risks of liver cirrhosis and initial hepatic dysfunction. Despite similar subsequent hepatic dysfunction rates, HBV-positive patients had significantly lower 5-year overall survival and progression-free survival. These results highlight HBV’s complex influence on HNSCC outcomes, urging tailored treatment approaches.

Although findings occasionally vary, the emerging evidence consistently points to a significant and complex association between HBV and HCV infections and the increased risk of various types of HNC, particularly oropharyngeal cancer. Further research is needed to resolve any conflicting results and to elucidate the underlying mechanisms driving these associations, which could inform targeted preventive strategies against these viral infections in the context of HNC.

## 8. The Intricacies of Co-Infections: Their Clinical Significance in Head and Neck Cancers

Co-infections with other pathogens can influence the oncogenic potential of viruses in the head and neck region, exacerbating cancer progression through various mechanisms. These interactions often lead to immunosuppression, chronic inflammation, and synergistic cellular damage, creating a more conducive environment for cancer development. The immunosuppression caused by HIV significantly increases the risk of HPV-related cancers at various anatomic sites, including HNCs [83]. These cancers tend to present at more advanced stages and exhibit more aggressive behavior. HIV-positive individuals are more likely to have persistent HPV infections due to their compromised immune systems, which fail to clear the virus effectively. This persistence increases the likelihood of oncogenic mutations and progression to malignancy. Additionally, while antiretroviral therapy improves overall immune function, it does not completely eliminate the increased cancer risk, highlighting the need for vigilant cancer screening and prevention strategies in this population [84].

Furthermore, a recent study by Salahuddin et al. [85] found that people living with HIV (PWH) and diagnosed with HNC have significantly poorer clinical outcomes compared to uninfected patients. HIV was an independent predictor of decreased overall survival, with PWH having a median survival of 39.1 months versus 100.8 months for uninfected patients. This difference was most pronounced in early-stage cancers and HPV-associated OPSCC. Additionally, tumors in PWH had lower CD8 T-cell infiltration and PD-L1 expression, which are associated with better outcomes. Additional investigation into HIV-associated HNC and customized treatment strategies for this demographic are essential.

Chronic bacterial infections can create an inflammatory environment that promotes viral persistence and oncogenesis [86]. A characteristic example is Helicobacter pylori, which is well-documented in the context of gastric cancer [87]. The oral cavity hosts a diverse microbiome, and the presence of multiple microbial species can interact synergistically to enhance oncogenesis. Specifically, chronic periodontitis, a common oral infection, has been associated with an increased risk of oral and oropharyngeal cancers. The persistent inflammation from periodontitis creates a favorable environment for oncogenic viruses by disrupting normal cellular homeostasis and weakening local immune responses [88]. Moreover, periodontitis leads to the release of free radicals and cytokines, mechanisms that are involved in carcinogenic and metastatic processes [89]. Additionally, chronic bacterial infections can induce epithelial–mesenchymal transition (EMT), a process by which epithelial cells acquire mesenchymal properties, enhancing their migratory and invasive capabilities. EMT is a critical step in cancer metastasis. Bacterial pathogens and the associated inflammatory responses can also activate signaling pathways such as NF-κB and STAT3, which are known to play roles in inflammation and cancer progression [90]. Regular dental check-ups, good oral hygiene, and timely treatment of periodontal diseases can also play a significant role in mitigating these risks.

Co-infection with multiple oncogenic viruses can also potentiate cancer risk. Several studies have documented the co-infection of HPV and EBV in HNCs [91,92]. These viruses can interact at the molecular level to enhance each other’s oncogenic potential. EBV’s role in modulating immune responses and promoting an inflammatory environment can complement HPV’s ability to evade immune detection and drive cell proliferation. The interaction between these viruses can lead to more aggressive tumor phenotypes and poorer clinical outcomes [93]. EBV and HPV co-infection significantly impacts oropharyngeal carcinomas, with global rates ranging from 15% to 20%, and higher rates (25% to 70%) in squamous cell carcinomas of the tonsils and base of the tongue [94]. Studies show that in vitro, co-infection enhances viral replication and alters viral cycle dynamics, with HPV potentially favoring EBV latency while EBV delays epithelial differentiation and promotes invasiveness, especially in cells expressing HPV-16 E6 and E7 oncogenes [44]. Research from Bosnia observed a 34.7% co-infection rate in HNSCC tissues, significantly correlating with advanced-stage disease, highlighting their collaborative role in cancer progression [95]. Further investigation is needed to fully elucidate EBV’s specific contribution to HPV-positive oropharyngeal cancers, potentially through mutual modulation of viral cycles.

Ursu et al. [96] explored oncogenic viruses in HNSCCs among Romanian patients. They tested 26 tumors for 67 viral agents and found that 88.5% were positive for one or more viruses, notably absent of HPV. Their study revealed a significant presence of herpesviruses and polyomaviruses in HPV-negative cases, with EBV-1, HHV-7, and MCPV frequently detected, suggesting their potential involvement in the pathogenesis of these cancers. Mulder et al. [97] examined the presence of HPV, EBV, and MCPV in HNSCC among non-smokers and non-drinkers (NSND). They found HPV in all oropharyngeal tumors and one oral tumor, and EBV in three nasopharyngeal tumors, with no MCPV detected. HPV or EBV positivity did not significantly affect 5-year disease-free or overall survival. The roles of HPV and EBV in specific HNSCC subtypes among NSND are limited in terms of survival outcomes, urging further research into virus-negative tumors for targeted therapies. Schindele et al. [98] analyzed the presence of HPV, EBV, CMV, and human adenovirus (HAdV) in LSCC, utilizing advanced molecular techniques across a cohort of 78 patients. Their findings, revealing EBV in 33% of tumor samples and lower frequencies of CMV and HAdV, point out the varying prevalence of these viruses in LSCC. The identification of high-risk HPV-16 in 9% of samples highlights its potential role, although p16 overexpression was observed more broadly in 14% of cases.

Understanding and addressing these interactions is crucial for developing comprehensive strategies to combat HNCs. Targeting the inflammatory pathways and managing chronic infections may reduce their incidence and progression.

## 9. Innovations in Viral Detection and Screening for Head and Neck Cancers

Innovations in viral detection and screening for HNCs have revolutionized surveillance and clinical outcomes. Cutting-edge techniques such as liquid biopsies and NGS offer enhanced sensitivity and non-invasive options for early diagnosis, treatment planning, and the monitoring of these malignancies. NGS has transformed the exploration of the molecular characteristics of HNSCC, significantly advancing precision medicine. By allowing comprehensive analysis of DNA and RNA in tumor tissues, NGS identifies viral sequences within the host genome, providing insights into integration sites, mutation profiles, and viral load. Its sensitivity enables the detection of low-abundance viral genomes, potentially identifying new contributors to oncogenesis. Customized NGS panels can screen for multiple viruses and mutations, offering a detailed molecular profile and insights into viral integration sites [99].

Dongre and colleagues [100] demonstrated the effectiveness of a customized targeted NGS panel in detecting specific mutations in long-term preserved, formalin-fixed paraffin-embedded (FFPE) tissue samples of HNSCC, even across samples stored for up to 17 years. In HPV-negative tumors, mutations were predominantly found in TP53, FAT1, and FLG genes, whereas HPV-positive tumors exhibited mutations in FLG, FAT1, and FGFR3 genes. The presence of these cancer-specific mutations correlated positively with features like extensive desmoplastic stroma and aggressive invasive fronts, and inversely with tumor differentiation. An analysis of TCGA data supported these findings, revealing poorer disease-free and overall survival outcomes in HNSCC patients with identified mutations. Despite advancements in precision medicine, treating HNSCC remains challenging due to its high mutation burden and inherent variability. Consequently, a combination of therapies is often necessary to optimize patient outcomes. This underscores the critical role of targeted NGS panels in identifying actionable mutations and guiding personalized treatment strategies for HNSCC.

Liquid biopsies detect circulating tumor DNA (ctDNA) and viral particles, offering real-time tumor genetic profiling for early HNC detection before symptoms, improving outcomes. They monitor treatment response, residual disease, and recurrence, enhancing personalized care [101]. Specifically, there is growing interest in using circulating plasma tumor HPV DNA for diagnosing and monitoring patients with HPV-associated OPSCC. Recent advancements, such as tumor tissue-modified viral (TTMV)-HPV DNA testing, combine identification of circulating HPV tumor DNA with tumor DNA fragment analysis, showing promising accuracy in clinical settings. In a retrospective observational cohort study of 399 patients, TTMV-HPV DNA testing demonstrated 91.5% sensitivity and 100% specificity for pre-treatment diagnosis of HPV-associated OPSCC, and 88.4% sensitivity and 100% specificity for surveillance of recurrences [102]. Further validation across diverse clinical contexts is needed before potential integration into standard practice guidelines.

A recent study led by Aye and colleagues [103] evaluated HPV-DeepSeek, a custom HPV whole-genome NGS liquid biopsy, for its diagnostic and prognostic utility in HPV-associated HNSCC. Their findings demonstrated that HPV-DeepSeek achieved superior sensitivity (98.7%), specificity (98.7%), and diagnostic accuracy (0.974) compared to traditional methods such as singleplex and multiplex ddPCR, HPV serology, and clinical diagnostic biopsy. Moreover, HPV-DeepSeek successfully identified high-risk HPV-16 single nucleotide polymorphisms, viral integration events, and PIK3CA mutations, highlighting its potential to advance diagnosis and prognostication in HPV-positive HNSCC.

Bhambhani et al. [104] recently demonstrated that transrenal ctDNA (TR-ctDNA) in the urine primarily consists of ultrashort fragments (<50 bp). Traditional ctDNA assays often miss these fragments, prompting the development of an ultrashort droplet digital PCR assay tailored for HPV-associated OPSCC. This advancement enables sensitive detection of cancer in urine samples, underscoring the potential of urine-based testing for convenient and non-invasive cancer diagnosis. Importantly, urine-based TR-ctDNA also shows promise for effective post-treatment surveillance in HPV-positive OPSCC, providing a less invasive method to monitor disease recurrence.

## 10. Conclusions

**Figure 1:**
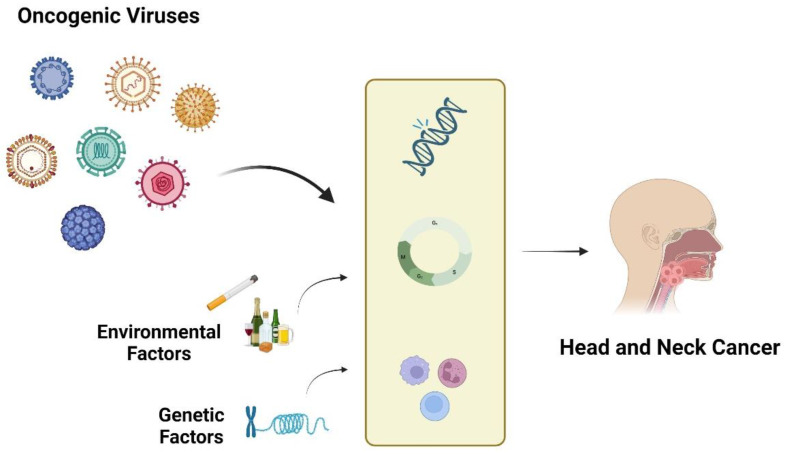
Illustration depicting the multifactorial pathogenesis of head and neck cancer, highlighting the role of oncogenic viruses in conjunction with environmental factors (such as tobacco smoke and alcohol consumption) and genetic factors (including inherited genetic mutations). The image shows how viruses can integrate into host DNA, disrupt normal cell cycle regulation, and evade immune surveillance. These mechanisms, combined with exposure to carcinogens and genetic predispositions, lead to malignant transformation and tumor development. Created with BioRender.com (accessed on 6 July 2024).

The complex viral landscape in HNCs plays a critical role in cancer development and progression. The interactions of these viruses with other environmental and genetic factors, coupled with their long latency periods, contribute to oncogenesis (Figure 1). Recent strides in identifying viral proteins and their interactions with host cells hold promise for innovative treatments and diagnostic biomarkers. Personalized approaches tailored to the specific viral profiles of HNCs offer the potential to significantly improve treatment outcomes. Continued research is indispensable to fully unravel these mechanisms and propel the development of effective strategies for managing viral-associated HNCs. In the post-COVID era, it is also possible that research will reveal other viruses with oncogenic potential or the ability to modify the immune system, further broadening our understanding of viral impacts on human health.

## Figures and Tables

**Table 1:** Overview of the identified oncogenic viruses as well as the viruses with potential oncogenic dynamic, their genome types (DNA or RNA), and the cancers in the head and neck region they are commonly associated with.

Virus	Genome Type	Associated Cancers in Head and Neck Region
Human Papillomavirus (HPV)	DNA	Oropharyngeal, laryngeal, oral cavity
Epstein–Barr Virus (EBV)	DNA	Nasopharyngeal, Burkitt & Hodgkin lymphomas
Human Herpesvirus 8 (HHV-8)	DNA	Kaposi’s sarcoma, primary effusion lymphoma, multicentric Castleman disease, laryngeal
Merkel Cell Polyomavirus (MCPV)	DNA	Merkel cell carcinoma, head & neck squamous cell, oral squamous cell carcinomas
Hepatitis B Virus (HBV)	DNA	Head & neck squamous cell carcinoma
Hepatitis C Virus (HCV)	RNA	Head & neck squamous cell carcinoma, oral cavity
Human Herpesvirus 1 (HHV-1)	DNA	Correlation with lip, oral cavity & pharynx
Cytomegalovirus (CMV)	DNA	Pro-oncogenic link with nasopharyngeal, protective effect with tumors of the lip/oral region & salivary glands
Human Herpesviruses 2, 3, 6, 7 (HHV-2, HHV-3, HHV-6, HHV-7)	DNA	Potential tumorigenic properties—not fully elucidated
Human Bocavirus (HBoV)	DNA	Potential involvement in tonsil squamous cell carcinoma
